# Exosome Release Is Regulated by mTORC1

**DOI:** 10.1002/advs.201801313

**Published:** 2018-12-11

**Authors:** Wenchong Zou, Mingqiang Lai, Yue Zhang, Lei Zheng, Zhe Xing, Ting Li, Zhipeng Zou, Qiancheng Song, Xiaoyang Zhao, Laixin Xia, Jian Yang, Anling Liu, Han Zhang, Zhong‐Kai Cui, Yu Jiang, Xiaochun Bai

**Affiliations:** ^1^ Department of Cell Biology Guangdong Provincial Key Laboratory of Bone and Joint Degenerative Diseases School of Basic Medical Sciences Southern Medical University Guangzhou 510515 China; ^2^ Department of Laboratory Medicine Nanfang Hospital Southern Medical University Guangzhou 510515 China; ^3^ Department of Orthopedics The Third Affiliated Hospital of Southern Medical University Guangzhou 510500 China; ^4^ Department of Hepatobiliary Surgery II Zhujiang Hospital Southern Medical University Guangzhou 510280 China; ^5^ Department of Developmental Biology School of Basic Medical Sciences Southern Medical University Guangzhou 510515 China; ^6^ Department of Biomedical Engineering Materials Research Institutes The Huck Institutes of The Life Sciences The Pennsylvania State University University Park PA 16802 USA; ^7^ Department of Biochemistry School of Basic Medical Sciences Southern Medical University Guangzhou 510515 China; ^8^ Department of Pharmacology and Chemical Biology University of Pittsburgh School of Medicine Pittsburgh PA 15261 USA

**Keywords:** exosomes, mTORC1, MVBs, rapamycin, TSC2

## Abstract

Exosomes are small membrane‐bound vesicles released into extracellular spaces by many types of cells. These nanovesicles carry proteins, mRNA, and miRNA, and are involved in cell waste management and intercellular communication. In the present study, it is shown that exosome release, which leads to net loss of cellular membrane and protein content, is negatively regulated by mechanistic target of rapamycin complex 1 (mTORC1). It is found that in cells and animal models exosome release is inhibited by sustained activation of mTORC1, leading to intracellular accumulation of CD63‐positive exosome precursors. Inhibition of mTORC1 by rapamycin or nutrient and growth factor deprivation stimulates exosome release, which occurs concomitantly with autophagy. The drug‐stimulated release is blocked by siRNA‐mediated downregulation of small GTPase Rab27A. Analysis of the cargo content in exosomes released from rapamycin‐treated cells reveals that inhibition of mTORC1 does not significantly alter its majority protein and miRNA profiles. These observations demonstrate that exosome release, like autophagy, is negatively regulated by mTORC1 in response to changes in nutrient and growth factor conditions.

## 1 Introduction

Exosomes are small membrane‐bound vesicles (40–200 nm in diameter) that are released to the extracellular space by most cell types under both physiological and pathological conditions.^1^ While initially considered as cell waste, these extracellular vesicles have been increasingly appreciated as mediators for intercellular communication.[[qv: 1b]] Exosomes contain signaling proteins, miRNA, mRNA, DNA, and lipids, which upon delivery are able to modulate the cellular activities of the recipient cells.^2^ Exosomes are found in many types of body fluids, including plasma and urine. The levels of exosomes are associated with numerous physiological and pathological activities and are often indicative of health, stress, and disease conditions.^3^ Recently, there is a growing interest in exploiting exosomes for diagnosis and therapeutic purposes.^4^ Nevertheless, despite many years of study, our understanding on the basic biology of exosomes remains limited. It is currently unclear whether formation and secretion of exosomes are regulated and whether exosomes alter their cargo content in response to changes in intracellular and extracellular conditions.[[qv: 1c]]

Exosomes are originated from intraluminal vesicles (ILVs) within endosomal compartments.^5^ Internalization of the plasma membrane by the process of endocytosis generates early endosomes. The subsequent inward budding of endosomal membrane produces ILVs within the endosomal compartments, resulting in formation of multivesicular bodies (MVBs). MVBs can either merge with lysosomes for cargo degradation or fuse with the plasma membrane to release ILVs into the extracellular space as exosomes. Formation of ILVs from endosomal membranes is regulated by the Endosomal Sorting Complex Required for Transport (ESCRT), which consists of four complexes, ESCRT‐0, I, II, and III. ESCRT‐0 controls cargo clustering, ESCRT‐I and II bud formation, and ESCRT‐III vesicle scission.[[qv: 3a]] During formation of ILVs, certain proteins from the plasma and endosomal membranes as well as the ESCRT complexes are selectively sequestered within ILVs, which often serve as markers for exosomes, including cluster of differentiation 63 (CD63), programed cell death 6‐interacting protein (ALIX), and tumor susceptibility gene 101 (TSG101). The docking and fusion of MVBs with the plasma membrane are regulated by two small GTPases of Rab family, Rab27A and Rab27B.^6^ As part of endosomal trafficking, fusion of MVBs with the plasma membrane and release of exosomes are critical not only for delivery of extracellular messengers but also for membrane homeostasis, lysosome function, and autophagy. A defect in the release is thus expected to have a profound impact on cell growth and metabolism.

Mechanistic target of rapamycin (mTOR) is a highly conserved Ser/Thr protein kinase that functions in two distinct complexes, mTOR complex 1 (mTORC1) and complex 2.^7^ mTORC1 is the rapamycin‐sensitive complex and involves in coordinating cellular metabolic activities in response to changes in growth factor, intracellular nutrient, and energy conditions. Its signaling activity is negatively regulated by the tuberous sclerosis complex genes 1/2 (TSC1/TSC2) complex formed by the products of TSC1/2.^8^ mTORC1 is a master regulator of autophagy, a controlled destructive mechanism that removes damaged or nonessential cellular components, including proteins and membranes, for recycling. Its inhibition, either by rapamycin or by nutrient deprivation, induces autophagy activation as part of stress responses essential for cell survival. Sustained mTORC1 activation, as it occurs in the TSC1/2‐deficient cells, results in downregulation of autophagy and, consequently, reduction in cell fitness.^9^ mTORC1 activity was typically assessed by phospho‐S6 (PS6) levels in western blot.

Regulation of autophagy represents a major mechanism whereby mTORC1 controls cellular metabolism through removal of cellular organelles and components. Similarly, exosome release also causes loss of cellular content by shedding membranes and cellular components into the extracellular space. In the present study, we show that this secretion‐dependent mechanism, like autophagy, is also regulated by mTORC1 in response to changes in nutrient and growth factor conditions.

## 2 Results

### 2.1 mTORC1 Activation Inhibits Exosome Release

**Figure 1:**
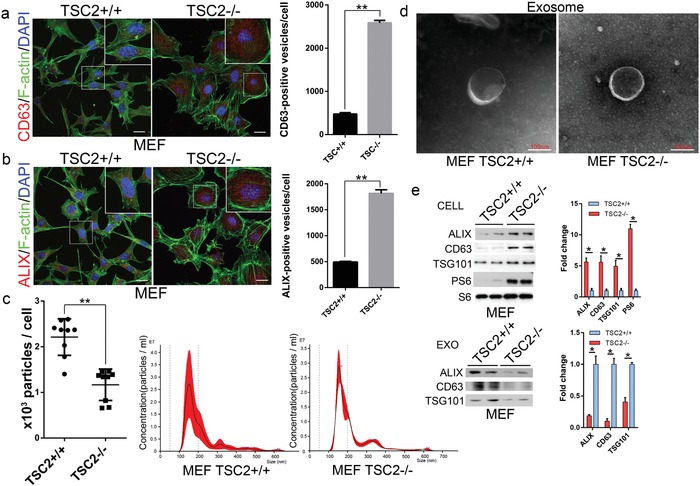
Constitutive activation of mTORC1 inhibits exosome release in cells. Immunofluorescent images of TSC2^+/+^ and TSC2^−/−^ MEFs stained with a) anti‐CD63 (red) or b) anti‐ALIX (red), together with phalloidin for F‐actin (green) and DAPI for nuclei (blue). Scale bars, 20 µm. Quantitative presentations of CD63 or ALIX‐positive vesicles in TSC2^+/+^ and TSC2^−/−^ MEFs are shown in the right panel. A total of 60 randomly selected cells from three independent experiments were analyzed. Isolated exosomes from TSC2^+/+^ and TSC2^−/−^ MEFs were examined by c) NTA and d) transmission electron microscopy. e) Cell lysates and exosomes isolated from culture media of TSC2^+/+^ and TSC2^−/−^ MEFs were assessed by western blot. Western blots were quantified. Three independent experiments were analyzed. **P < 0.01.

While studying exosome levels in aged mice, we serendipitously discovered that mouse embryo fibroblasts (MEFs) from TSC2 null mice exhibited a strong staining of CD63‐containing vesicular structures in comparison with their cognate wild type controls. As CD63 is normally concentrated on ILVs, this observation indicates an abnormal accumulation of the intraluminal vesicles in the TSC2‐deficient cells (**Figure** **1**a). To confirm the CD63‐positive vesicular structures are ILVs, we stained TSC2^−/−^ MEFs and their wild‐type controls with another ILV marker, ALIX. We found that the TSC2‐deficient MEFs contained many more ALIX‐positive vesicular structures than controls (Figure 1b).

The abnormal accumulation of ILVs in the TSC2‐deficient cells could be resulted from overproduction of ILVs or blockage in their release. To distinguish these two possibilities, we compared the levels of several commonly used ILV/exosome markers in total cell lysates and the exosomes released into the culture media from TSC2^−/−^ and TSC2^+/+^ MEFs. TSC1 and TSC2 regulate the actin cytoskeleton in a differential manner.^10^ TSC2 modulates actin cytoskeleton and focal adhesion through TSC1‐binding domain and the Rac1 GTPase, resulting in different morphology of MEFs isolated from TSC2^+/+^ and TSC2^−/−^. The released exosomes in culture media after a given amount of time were isolated by differential centrifugation as described previously.^11^ Examination of the isolated exosomes using nanoparticle tracking analysis (NTA) showed that the exosomes from TSC2^−/−^ and wild‐type MEFs had a similar size distribution (Figure 1c). They also displayed a similar morphology and size (Figure 1d). However, a significant lesser amount of exosomes was recovered from the culture media of TSC2^−/−^ MEFs than from wild‐type MEFs (Figure 1c). Western blot analysis revealed that the amounts of exosome marker proteins, including CD63, ALIX, and TSG101, in the total exosomes isolated from culture media of TSC2^−/−^ MEFs were drastically lower, whereas those in total cell extracts were much higher than their wild‐type counterparts (Figure 1e). These findings suggest that the intracellular accumulation of ILVs in TSC2^−/−^ MEFs is caused by a blockage in their release.

TSC2 normally functions in complex with TSC1 to elicit its negative activity on mTORC1.^8^ To determine whether TSC1 is also involved in exosome release, we examined the effect of TSC1 downregulation on the process. We found that knockdown of TSC1 with siRNA in HEK293 and HeLa cells resulted in an increased intracellular accumulation of CD63‐positive vesicular structures (Figure S1a,b, Supporting Information). The amounts of exosome marker proteins, CD63, ALIX, and TSG101, were significantly increased in cell lysates but reduced in total exosomes isolated from culture media when TSC1 was downregulated by siRNA (Figure S1c,d, Supporting Information). These findings demonstrate that TSC1, like TSC2, is required for exosome release. From CD63 immunogold staining on the exosomes collected from MEFs with transmission electron microscope (TEM), we can obviously observe the gold‐labeled exosomes (Figure S1e, Supporting Information). We believe that the CD63 immunofluorescence staining images could identify the exosomes.

### 2.2 Inhibition of mTORC1 by Rapamycin Stimulates Exosome Release

**Figure 2:**
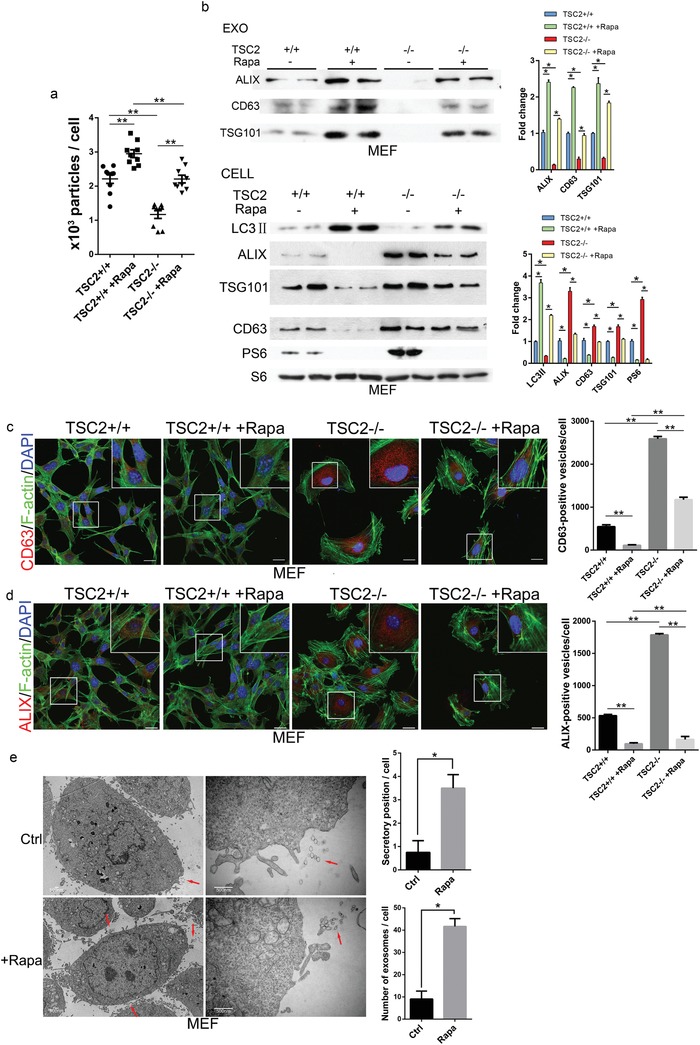
Inhibition of mTORC1 stimulates exosome release. a) TSC2^+/+^ and TSC2^−/−^ MEFs were treated with rapamycin (1.0 × 10^−7^ m) or vehicle control for 24 h. The numbers of exosomes isolated from culture media at the end of rapamycin treatment were assessed by NTA. b) The levels of exosome marker proteins in cell lysates (Cell) and isolated exosomes (Exo) were examined by western blot. Western blots were quantified. mTORC1 activity in cell extracts was assayed based on the levels of PS6. Three independent experiments were analyzed. Cells were fixed and stained with c) anti‐CD63 (red) or d) anti‐ALIX (red), together with phalloidin for F‐actin (green) and DAPI for nuclei (blue). Stained cells were examined by confocal microscopy. Scale bars, 20 µm. Right panels are quantitative presentations of the numbers of CD63 or ALIX‐positive vesicles in rapamycin and mock‐treated cells. For each treatment and staining, a total of 60 randomly selected cells from three independent experiments were analyzed. e) Transmission electron microscopy analysis of MEF cells treated with rapamycin (1.0 × 10^−7^ m) or vehicle control for 24 h. The released exosomes in the extracellular space were marked by red arrows. Right panels are quantitative presentations of the numbers of exosomes in the extracellular space. Scale bars, 500 nm.

TSC1 and TSC2 are negative regulators of mTORC1 and their downregulation causes mTORC1 activation. To determine whether the hyperactive mTORC1 in TSC2^−/−^ MEFs is the cause for the blockage in exosome release, we examined the effect of rapamycin on the release. Both TSC2^−/−^ and TSC2^+/+^ MEFs were treated with rapamycin or vehicle control phosphate buffer saline (PBS) and exosomes released into the culture media at the end of treatment were collected. NTA revealed a large increase in the amount of exosomes from media of rapamycin‐treated cells in comparison with those from mock‐treated cells (**Figure** **2**a). The drug‐stimulated release was confirmed by an increase in the amounts of CD63, ALIX, and TSG101 in the total exosomes isolated from culture media and a concomitant decrease of their levels in cell extracts (Figure 2b). The drug‐induced exosome release is concomitant with activation of autophagy, which was manifested by an increased level of autophagy marker, light chain 3‐II (LC3II) (Figure 2b). Rapamycin also caused a sharp reduction in the amounts of intracellular CD63 and ALIX‐positive vesicular structures (Figure 2c,d). Transmission electron microscopy revealed a strong increase in exosome accumulation in the extracellular space of the MEFs treated with rapamycin (Figure 2e). The stimulating effect of rapamycin on exosome release was also observed in HEK293 (Figure S2a–c, Supporting Information) and HeLa cells (Figure S3a–d, Supporting Information). Living cell imaging and NTA examination of HeLa cells expressing green fluorescent protein (GFP)‐CD63 showed that rapamycin induced a time‐dependent release of exosomes (Movies S1 and S2 and Figure S3e, Supporting Information). Taken together, these findings demonstrate that exosome release is suppressed by sustained mTORC1 activation but simulated by mTORC1 inhibition, suggesting a negative role for mTORC1 in the process.

### 2.3 Amino Acids or Serum Starvation Stimulates Exosome Release in Cultured Cells

**Figure 3:**
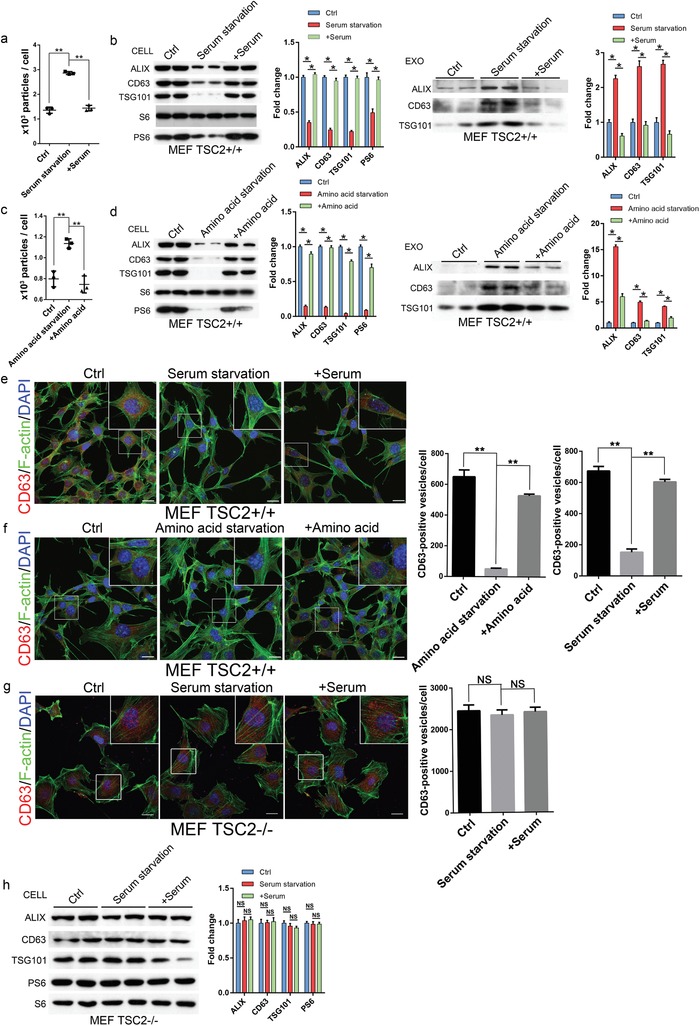
Serum or amino acid deprivation stimulates exosome release. a,b) TSC2^+/+^ MEFs were cultured in serum‐containing medium for 16 h followed by shifting to serum‐deprived medium. After incubation for 16 h, the cells were then shifted back to serum‐containing medium for additional 16 h. The numbers of exosomes isolated from the media before and after each shift were determined by NTA (a). The levels of exosome marker proteins in cell extracts (Cell) and released exosomes (Exo) before and after each shift were determined by western blot. Western blots were quantified. The mTORC1 activity in the cells was monitored by levels of PS6 (b). Three independent experiments were analyzed. c,d) TSC2^+/+^ MEFs were cultured in amino acid‐containing medium for 2 h before being shifted to amino acid‐free medium. After incubation for 2 h, the cells were then shifted back to amino acid‐containing medium for additional 2 h. The numbers of released exosomes before and after each shift were determined by NTA (c) and levels of exosome marker proteins in cell lysates (Cell) and released exosomes (Exo) by western blot. Three independent experiments were analyzed (d). e,f) Cells before and after each shift were fixed and stained with anti‐CD63 (red), phalloidin for F‐actin (green), and DAPI for nuclei (blue). Scale bars, 20 µm. Right panels are quantitative presentations of the numbers of CD63‐positive vesicles in the cells. For each shift point, a total of 60 randomly selected cells from three independent experiments were analyzed. g,h) TSC2^−/−^ MEFs were subjected serum deprivation and repletion as described above. The treated cells were fixed and stained as in (e). Scale bars, 20 µm. The right panel shows the quantitative presentations of the numbers of CD63‐positive vesicles in the cells. For each shift point, a total of 60 randomly selected cells from three independent experiments were analyzed (g). The levels of exosome marker proteins and mTORC1activity in cell lysates were determined by western blot (h). **P < 0.01.

The finding that exosome release is controlled by mTORC1 suggests that the process is likely to be responsive to changes in nutrient and growth factor conditions. To confirm this notion, we subjected TSC2^+/+^ MEFs to serum deprivation for 16 h or amino acid limitation for 2 h. NTA analysis showed that serum starvation, which reduced mTORC1 activity, strongly stimulated exosome release from the cells (**Figure** **3**a). Western blot showed that the amounts of CD63, ALIX, and TSG101 in the exosomes isolated from culture media were increased, whereas those in cell extracts were reduced (Figure 3b). Similar results were found in cells underwent amino acid limitation (Figure 3c,d). Consistent with the reduced intracellular levels of the exosome marker proteins, intracellular CD63 and ALIX‐positive vesicular structures were also reduced by serum deprivation or amino acid limitation (Figure 3e,f). The serum deprivation or amino acid limitation induced increase in exosome release was reversed when the stressed cells were shifted back to normal media. In contrast, serum deprivation failed to induce exosome release in TSC2^−/−^ MEFs, in which the mTORC1 activity was largely insensitive to the deprivation (Figure 3g,h). The nutrient and growth factor sensitive regulation of exosome release was further confirmed in HeLa cells (Figure S4a–c, Supporting Information). These findings demonstrate that exosome release, like autophagy, is responsive to changes in nutrient and growth factor conditions.

### 2.4 mTORC1 Controls Exosome Release In Vivo

**Figure 4:**
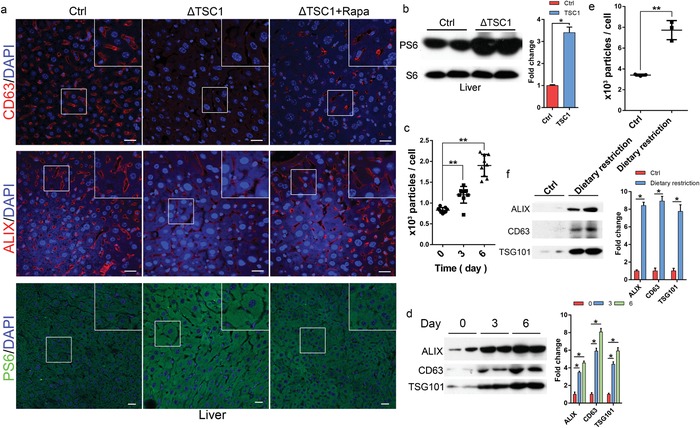
mTORC1 regulates exosome release in vivo. Three‐month‐old mice (*n* = 5) with liver cell‐specific TSC1 deletion (TSC1KO) were administrated with rapamycin (2.5 mg kg^−1^ d^−1^) or drug vehicle for 2 months. a) Liver tissues were collected and stained with anti‐CD63 (red), ALIX (red), or PS6 (S235/236) (green), together with DAPI (blue). Scale bars, 20 µm. b) Liver tissues were lysed and mTORC1 activity was determined by PS6 levels. C57BL/6 mice were administrated with rapamycin (2.5 mg kg^−1^ d^−1^) or drug vehicle at indicated time‐points. Serum exosomes were isolated from the treated animals and subjected to c) NTA or d) western blot analysis. Western blots were quantified. C57BL/6 mice were diet‐restricted for 48 h, and serum exosomes isolated from treated and control animals were analyzed by e) NTA or f) western blot. **P < 0.01.

We next evaluated whether mTORC1 modulates exosome release in vivo. It has been shown that liver cells release a large amount of exosomes into the extracellular space and circulation.^12^ The release is influenced by disease conditions, including alcoholic liver disease, viral hepatitis, and hepatocellular carcinoma.^13^ To determine whether mTORC1 activity modulates liver exosome release, we generated mice with hepatocyte‐specific mTORC1 activation by crossing TSC1^flox/flox^ line with a transgenic line expressing cyclization recombination enzyme (CRE) under the albumin promoter (Alb‐Cre). We found that the liver‐specific deletion of TSC1 led to constitutive activation of mTORC1 in liver cells and drastic reduction of CD63 and ALIX‐positive staining vesicles in the extracellular space of the liver tissues (**Figure** **4**a,b). The effect was liver specific, as other organs, such as the kidney and heart, were not affected (Figure S5a–c, Supporting Information). Treating the deletion animals with rapamycin reduced mTORC1 activity and increased CD63 and ALIX‐positive staining vesicles in the extracellular space of liver tissues (Figure 4a). Rapamycin also caused a marked and time‐dependent increase in plasma exosome level in wild‐type C57BL/6 mice, as determined by analysis of the numbers and protein levels of the exosomes isolated from the plasma of the treated animals (Figure 4c,d). As cellular mTORC1 activity is affected by nutrient intake in animals, we next examined the effect of dietary restriction on plasma exosome levels in wild‐type C57BL/6 mice. Upon subjected mice to fasting for 48 h, blood plasma was collected from treated and control animals and exosomes isolated. NTA analysis revealed that dietary restriction induced a strong increase in the plasma exosome level (Figure 4e). Western blotting analysis also showed an increase in the amounts of exosome marker proteins in the exosomes isolated from the plasma of the diet‐restricted mice in comparison with that from untreated animals (Figure 4f). These observations demonstrate that in vivo exosome release is regulated by mTORC1 and food intake.

### 2.5 Inhibition of mTORC1 Induces Exosome Release without Significantly Altering Its Cargo Content

**Figure 5:**
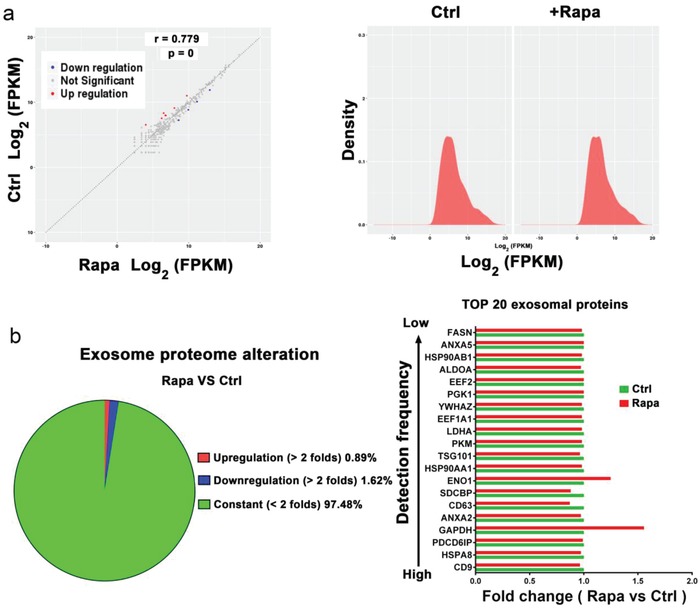
Inhibition of mTORC1 induces exosome release without significantly altering its cargo content. HeLa cells were treated with rapamycin (1.0 × 10^−7^ m) or drug vehicle for 24 h. Exosomes isolated from culture media were subjected to miRNA sequence analysis. a) (left panel) The correlation of miRNA expression between rapamycin (Rapa) or mock‐treated (Ctrl) cells is shown. Right panel: The distribution of miRNA expression (Rapa vs Ctrl). b) Protein profile of the isolated exosomes was analyzed by iTRAQ‐coupled 2D LC‐MS/MS. The percentage of unchanged, upregulated (more than two folds), or downregulated (more than two folds) proteins (Rapa vs Ctrl) is shown by a sector graph (left panel). The fold changes of the top 20 most often identified exosome proteins (Rapa vs Ctrl) based on statistical data from ExoCarta are shown (right panel). Green bar, con. Red bar, rapamycin.

Exosomes contain cell‐specific cargoes of protein, mRNA, and miRNA, which can function as signaling molecules for intercellular communication.[[qv: 1c,2]] To determine whether mTORC1 regulates the cargo content of exosomes, we performed miRNA sequencing and proteomic analysis on exosomes released from HeLa cells treated with rapamycin or vehicle control. We found that although rapamycin treatment markedly increased exosome release (Figure 1, 2), the miRNA and protein content from the treated and mock‐treated cells are largely indistinguishable. We identified only a few miRNAs and proteins that were differentially expressed between the paired samples (**Figure** **5**a,b and **Table** **1**, and Table S1 in the Supporting Information ); miRNA sequencing data have been deposited in the GEO database under accession code GSE114235. This finding indicates that mTORC1 controls exosome release rather not specific cargo selection.

**Table 1:** Upregulated or downregulated micro‐RNAs in exosomes from HeLa cells treated with rapamycin (1.0 × 10^−7^ m) for 24 h

miRNA	log_2_FC (HeLa + Rap/HeLa)	*P* value	*Q* value	Up/down
hsa‐miR‐10b‐3p	2.544454571	0.001488068	0.020535335	Up
novel.14	1.838540205	2.89E‐06	0.000144859	Up
hsa‐miR‐409‐3p	1.322099051	0.002779664	0.032646269	Up
novel.3	1.260082997	0.001065918	0.017829896	Up
novel.51	1.255952792	6.62E‐09	9.14E‐07	Up
novel.22	1.161625241	0.002220226	0.02918011	Up
novel.21	1.116765975	6.21E‐05	0.002016604	Up
hsa‐let‐7c‐5p	−1.066246768	2.96E‐05	0.00116858	Down
hsa‐miR‐941	−1.072426993	0.00014307	0.003823111	Down
hsa‐miR‐328‐3p	−1.103001842	0.000738548	0.013150926	Down
hsa‐miR‐455‐5p	−1.354054825	0.001621181	0.021826637	Down

novel refers to pre‐unidentified sequences.

### 2.6 mTORC1 Regulates Exosome Release through a Rab27A‐Dependent Mechanism

**Figure 6:**
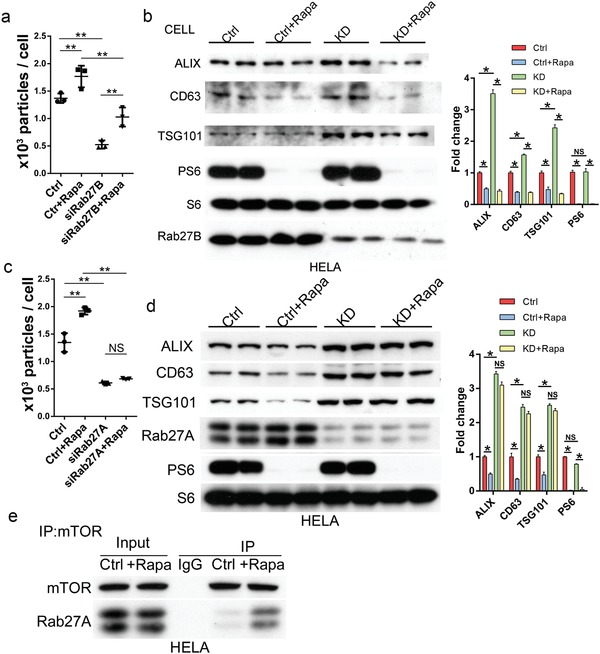
mTORC1 regulates exosome release through a Rab27A‐dependent mechanism. a,b) HeLa cells expressing Rab27B siRNA were treated with rapamycin (1.0 × 10^−7^ m) or vehicle control for 24 h. The numbers of exosomes isolated from culture media at the end of the treatment were assessed by NTA (a) and the levels of exosome marker proteins in cell lysates by western blot. Western blots were quantified (b). c,d) HeLa cells transfected with Rab27A siRNA were treated with rapamycin (1.0 × 10^−7^ m) or vehicle control for 24 h. The numbers of exosomes isolated from culture media were assessed by NTA (c) and the levels of exosome marker proteins in cell lysates by western blot. Three independent experiments were analyzed (d). e) HeLa cells were treated with rapamycin (1.0 × 10^−7^ m) or vehicle control for 24 h. Cell lysates from the treated cells were immunoprecipitated with anti‐mTOR antibody and the amounts of Rab27A co‐purified with mTOR were determined by western blot.

Rab27A and Rab27B are two closely related Rab small GTPases that have been shown to play a critical role in exosome secretion in many types of cells.^14^ To determine how mTORC1 controls exosome release, we examined the role of the two Rab proteins in the process. We found that knockdown of Rab27B in HeLa cells by siRNA reduced exosome levels in culture media and increased intracellular levels of exosome markers CD63, ALIX, and TSG101 (**Figure** **6**a,b). This observation is consistent with a positive role of the small GTPase in exosome release. However, the siRNA‐mediated downregulation of Rab27B failed to block rapamycin‐stimulated exosome release (Figure 6a,b), indicating that Rab27B is not an effector of mTORC1 in controlling exosome release. Knockdown of Rab27A by siRNA also reduced exosome release, as manifested by reduced levels of exosomes in culture media and increased exosome marker proteins in cell extracts (Figure 6c,d), confirming the positive role of the small GTPase in the process. However, unlike Rab27B downregulation, knockdown of Rab27A inhibited rapamycin‐induced exosome release and increased intracellular accumulation of exosome marker proteins (Figure 6c,d). These findings indicate that mTORC1 controls exosome release via a Rab27A‐dependent mechanism. In support of this view, we found that Rab27A was able to associate with mTOR and that the association was enhanced by rapamycin treatment (Figure 6e).

## 3 Discussion

Exosome release and autophagic flux are two connected processes that cause loss of cellular membrane and protein content. Previous studies have shown that autophagy is tightly controlled by mTORC1 in response to nutrient and growth factor conditions.^15^ In the present study, we show that exosome release, like autophagic flux, is also regulated by mTORC1. The effect of mTORC1 on exosome release is reminiscent of that on autophagy in that active mTORC1 inhibits the release whereas inactive mTORC1 promotes the process. Our study, thus for the first time, demonstrates that exosome release is regulated by mTORC1 and sensitive to changes of amino acid and growth factor conditions.

The finding that TSC1 and TSC2‐deficient cells accumulate CD63‐positive vesicular structures suggests that mTORC1 acts at a late stage of exosome biogenesis, likely at the stage of docking/fusion of MVBs with the plasma membrane. This notion is supported by the involvement of Rab27A in mTORC1‐mediated exosome release. This small GTPase is known to function in MVBs docking at the plasma membrane.[[qv: 14a]] In addition, the finding that inhibition of mTORC1 increases exosome release without significantly altering its cargo content also indicates that mTORC1 controls exosome release but not formation. Although a few of protein and miRNA profiles were observed with some relatively high fold changes (Table 1 and Table S1, Supporting Information), the majority cargo content was not significantly altered. The functional assay of isolated exosomes after the treatment, particularly of those newly identified miRNAs, will be further investigated to extend the knowledge on the underline mechanism. On the other hand, since MVBs can fuse either with lysosome to deliver ILVs and their content for degradation or with the plasma membrane to release ILVs into extracellular space as exosomes,[[qv: 1c,5]] it is likely that inactivation of mTORC1 shunts MVBs to fusion with the plasma membrane at the expense of lysosomal delivery.

Both autophagy and exosome release are involved in maintenance of cellular protein, RNA, and membrane homeostasis.[qv: 2,3] While autophagy salvages membranes and proteins for recycling their constituents, exosome release results in net loss of membrane and proteins. A balance between the two processes is thus likely a key for keeping cell fitness under various conditions.^16^ The involvement of mTORC1 in both processes provides a means for coordinating their activities to maintain such a balance. In addition to their role in discharge of excessive membrane and secretion of damaged proteins and RNAs, exosomes also carry many types of signaling molecules and function as mediators for intercellular communication.^2^, ^17^ The increased release of exosomes when mTORC1 is inhibited by stress conditions may serve as a way for communicating with other cells to orchestrate a systemic response. A recent finding that the exosome cargo exerts beneficial effects on nutrient starved cells is consistent with this notion.^18^ Conversely, sustained mTORC1 activation prevents release of exosomes, causing accumulation of ILVs. Such an abnormality is likely to perturb the endosomal network and reduce exosome‐mediated secretion of signaling molecules. Defects in exosome release may thus contribute to the pathophysiological conditions in tuberous sclerosis complex syndrome and other diseases associated with hyperactive mTORC1.

Recently, there is a growing interest in exploiting exosomes for therapeutic purposes, such as targeted delivery of drugs, inhibitors, miRNAs and siRNAs, and harnessing the regenerative properties of stem cell derived exosomes.^19^ A major technical hurdle for these applications is to produce sufficient exosomes with stable and consistent properties.^20^ In this regard, our finding that inhibition of mTORC1 by rapamycin or nutrient starvation stimulates exosome release without significantly altering the content of exosomes offers a unique way to overcome the hurdle. It supports a potential use of rapamycin or starvation as a simple and effective method to improve production of exosomes.

In summary, our study shows that exosome release is regulated by mTORC1 in response to changes in nutrient and growth factor conditions and that mTORC1 activation inhibits exosome release in cultured cells and in vivo. This newly discovered role of mTORC1 places this master regulator of growth in another cellular content management mechanism, which functions together with autophagy to remove unwanted proteins and membranes. The concurrent regulation of these two processes by mTORC1 allows a cell to coordinate waste management and recycling, which is likely to play a key role in cell fitness under various adverse conditions. In addition, increased exosome release has been described in cells exposed to stress stimuli like glucose starvation and hypoxia.^21^ In light of our findings, it is of interest to investigate whether the increase in exosome release under those stress conditions is also regulated by mTORC1.

## 4 Experimental Section

*Cell Culture*: TSC2^+/+^ and TSC2^−/−^ MEFs and HeLa and HEK293 cells were cultured in Dulbecco's modified Eagle's medium (DMEM, Corning, New York, NY, USA) containing 10% fetal bovine serum (FBS, Gibco, Waltham, MA, USA). Wild‐type mouse embryonic fibroblasts (TSC2^+/+^ MEFs) and TSC2‐depleted mouse embryonic fibroblasts (TSC2^−/−^ MEFs) were kindly provided by Dr. David J. Kwiatkowski (Brigham and Women's Hospital, Boston, MA, USA). TSC2^−/−^ MEFs were immortalized by p53 deletion. The MEFs passage number was about 25‐27 generations. No surface coating material was used in the study, and MEFs were cultured in Eppendorf (0030700104, Hamburg, Germany) dishes. pCMV6‐GFP‐CD63 was purchased from Obio Technologies (Shanghai, China) and introduced into HeLa cells for stable expression of GFP‐CD63. Primary antibodies used in the study were anti‐CD63 (#ab193349), TSG101(#ab83), and ALIX (#ab117600) from Abcam (Cambridge, UK); anti‐Phospho‐S6 (#4858), LC3II (#3868), and mTOR (#2972) from Cell Signaling Technology (Boston, MA, USA); anti‐S6 (sc‐74459) from Santa Cruz Biotech (Santa Cruz, CA, USA); and anti‐Rab27A (#17817‐1‐AP) and Rab27B (#13412‐1‐AP) from ProteinTech (Shanghai, China). 4′,6‐diamidino‐2‐phenylindole (DAPI) for nucleus staining was from Fisher Scientific (#D1306, Waltham, MA, USA). F‐actin (#A12379) was obtained from Invitrogen (Waltham, MA, USA). Rapamycin (HY‐10219) was purchased from MCE (Monmouth Junction, NJ, USA). Cells were incubated with rapamycin (1.0 × 10^−7^ m) or vehicle control (PBS) for 24 h. PBS was purchased from Gibco (10010023, Waltham, MA, USA).

*Animals*: Animal experiments were approved by the Ethical Committee for Animal Research of Southern Medical University and conducted according to the state guidelines from the Ministry of Science and Technology of China. The TSC1^flox/flox^ (Stock No.: 005680) and Alb‐Cre (Stock No.: 003574) mouse strains were purchased from Jackson Laboratory (Las Vegas, NV, USA). To generate liver‐specific TSC1 deletion mice, TSC1^flox/flox^ mice were crossed with Alb‐Cre mice. C57BL/6 mice (newborn or 4–5 weeks old) were purchased from the Laboratory Animal Center of Southern Medical University (Guangzhou, China). Both male and female mice were used. The mice were housed in plastic cages at a controlled temperature of 22 ± 1 °C on a 12 h light/12 h dark cycle with lights on from 0600 to 1800 h. Standard rodent chow and water were provided ad libitum throughout the entire study period. For rapamycin treatment experiment, mice were treated with the drug by intragastric administration at 2.5 mg kg^−1^ d^−1^ for up to 6 d. For dietary restriction experiment, mice were kept on fasting for 48 h, during which time water was provided normally.

*Exosome Isolation*: Cells were cultured in medium supplemented with 10% FBS with exosome depleted by overnight centrifugation at 100 000 g. Medium was collected after the cells were cultured for 20 h or indicated time and subjected to centrifugation at 320 g for 10 min followed by centrifugation at 2 000 g for 15 min to remove debris and dead cells. The medium was further centrifuged at 10 000 g for 30 min at 4 °C (Beckman Coulter Optima L‐100 XP, Beckman Coulter). The supernatant was centrifuged at 100 000 g for 70 min at 4 °C. The resulting pellet, which contained exosomes, was washed once in PBS and collected by centrifugation at 100 000 g for 70 min.

*Density Gradient Centrifugation*: To generate a discontinuous density gradient, 3 mL of 40%, 20%, and 10% w/v OptiPrepiodixanol solution (Axis‐Shield, Dundee, Scotland), respectively, and 2.5 mL of 5% iodixanol solution were sequentially layered in an ultracentrifuge tube. Exosomes isolated by differential centrifugation were overlaid on the discontinuous gradient and centrifuged at 110 000 g for 16 h. An aqueous of 1 mL was collected from the top of the gradient. Each sample was diluted with PBS and centrifuged at 110 000 g for 70 min. The resultant pellet was resuspended in PBS.

*Isobaric Tags for Relative and Absolute Quantification (iTRAQ), 2D Liquid Chromatography‐Tandem Mass Spectrometry (LC‐MS)/MS, and Data Analysis*: iTRAQ labeling was conducted according to the manufacturer's instructions (Applied Biosystems, Foster city, CA, USA). Briefly, exosomal proteins from the control or rapamycin‐treated HeLa cells were harvested, reduced, alkylated, and digested with trypsin overnight at 37 °C before being labeled by iTRAQ. The control group was labeled with reporter tag 116, while rapamycin‐treated group with reporter tag 118. The labeled peptides were pooled and desalted with Sep‐Pak Vac C18 cartridges (Waters, Milford, MA, USA) followed by separation and analysis with a nano‐Acquity ultra performance LC system (100 µm × 100 mm C18BEH column) (Waters, Milford, MA, USA) coupled to a Q‐Exactive mass spectrometer (Thermo Fisher Scientific, Waltham, MA, USA). All raw files were analyzed using the Thermo Proteome Discoverer (1.3.0.339) software. The files were searched using the Mascot (2.2.04) search engine against the UniProt human (72 390 entries) and decoy human databases with a strict false discovery rate (FDR) of 0.01. The fold change threshold was set at 2.0. A *P* value (*P* > 0.05) criterion (between untreated/control ratios and sample/control ratios) was used to determine differentially expressed proteins.

*Immunogold Staining*: Cells were cultured and treated as described above. For immunoelectron microscopy, MEFs were fixed using 2% paraformaldehyde and 0.2% glutaraldehyde in PBS buffer (pH 7.4) at 37 °C for 2 h. Cell samples were pelleted and fixed for 1 h in 2.5% glutaraldehyde in phosphate buffer (0.1 m) at room temperature. Then, cells were subjected to blocking buffer and incubated overnight at 4 °C in droplets of mouse anti‐CD63 (1:500, #ab193349) from Abcam (Cambridge, UK). At the end of incubation, the cells were washed thrice to remove excess primary antibody and incubated in blocking buffer and then incubated with droplets of the gold‐labeled (10 nm) goat anti‐mouse IgG (1:50; Sigma‐Aldrich, G7652) for 2 h at room temperature. After several washings in PBS, the fixed samples were embedded in resin, thin sectioned (40 nm) and stained with uranyl acetate. Cells were loaded onto glow‐discharged carbon‐coated copper grids. The stained cell samples were observed using H‐7600 TEM (Hitachi, Japan) at an acceleration voltage of 80 kV.

*miRNA Sequencing*: The total RNA extraction of the sample was carried out using the miRNeasy Serum/Plasma Kit (Cat #217184, Qiagen) according to the standard operating procedures provided by the manufacturer. The extracted total RNA was subjected to electrophoresis on an Agilent 2100 Bioanalyzer (Agilent Technologies, Santa Clara, CA, USA). The sequencing RNA library was constructed by performing a 3′‐end linker, a 5′‐end linker, reverse transcription, amplification, cDNA library size selection, and purification steps on Total RNA. The Qubit 2.0 Fluorometer was employed to detect the concentration of the library and the size of the library was detected using the Agilent 2100. Cluster generation and first‐stage sequencing primer hybridization were performed on the cBot of the Illumina HiSeq sequencer from Shanghai Biotechnology Corporation according to the corresponding procedure in the cBot User Guide. Then the sequencing reagent was prepared following the Illumina User Guide and the flow cell carrying the cluster was loaded in the machine. Single‐end sequencing was performed using the single‐read program. The sequencing process was controlled by the data collection software provided by Illumina that performs real‐time data analysis.

*Exosome Isolation from Animal Plasma*: Blood was drawn from animal hearts and allowed to clot for 3 h at room temperature followed by incubation at 4 °C for 2 h, and serum was obtained by centrifugation at 400 g for 10 min at 4 °C. Cell debris was removed from the serum by centrifugation at 3 000 g for 15 min at 4 °C. The clarified serum was centrifuged at 100 000 g for 70 min at 4 °C. The exosome pellet was washed once with PBS and pelleted again by centrifugation at 100 000 g for 70 min before being resuspended in radioimmunoprecipitation assay buffer (RIPA) buffer.

*Nanoparticle Tracking Analysis*: Isolated exosomes were examined with the Nanosight N3000 system for numbers and size distribution and the data were analyzed by NTA 3.2 Dev Build 3.2.16.

*Gene Silencing*: Gene‐specific siRNAs and scrambled control siRNAs were transfected into HEK293 and HeLa cells using Lipofectamine 2000 (Invitrogen, Carlsbad, CA, USA). 48 h after transfection, cells were switched to exosome‐depleted medium for 20 h before analysis. The sequences of the siRNAs used were: *TSC1*, 5‐GCACUCUUUCAUCGCCUUUTT‐3 and 5‐CCAAAUCUCAGCCCGCUUUTT‐3, *Rab27A*, 5‐CAAGAGAGGUUUCGUAGCUUGACAA‐3 and 5‐CCAACUACAAAUGCAUGCAUAUUGU‐3, *Rab27B*, 5‐GGGACCAAAUGGAUCAUCAGGGAAA‐3 and 5‐GAGCCAACUGCAAGCAAACGCUUAU‐3, Negative control, 5‐UUCUCCGAACGUGUCACGUTT‐3 and 5‐ACGUGACACGUUCGGAGAATT‐3. The cells (2 × 10^5^) were transfected with 50 nmol L^−1^ of TSC1 siRNA, 50 nmol L^−1^ of Rab27A siRNA, 50 nmol L^−1^ of Rab27B siRNA, or 50 nmol L^−1^ of scrambled siRNA for the control group.

*Fluorescence Microscopy*: Cells were fixed with 4% paraformaldehyde and stained with the indicated primary antibodies followed by fluorescent‐dye conjugated secondary antibodies (Molecular Probes, Waltham, MA, USA). Images were obtained using a confocal laser scanning microscope (Olympus FV1200, Tokyo, Japan). Nuclei were stained with DAPI.

*Western Blotting*: Cells or exosomes were lysed in RIPA buffer containing 5.0 × 10^−2^ m Tris‐HCl pH 8, 0.15 m NaCl, 1% Triton X‐100, 0.1% sodium deoxycholate, 0.1% sodium dodecyl sulfate (SDS), and 1x protease inhibitor cocktail (Roche, Basel, Switzerland). Lysates were boiled in 1 x SDS sample buffer. Proteins were separated on SDS polyacrylamide gel electrophoresis (PAGE) and transferred to a nitrocellulose membrane for blotting with antibodies.

*Electron Microscopy*: Exosomes suspended in PBS were loaded onto glow‐discharged carbon‐coated copper grids and stained with 1% w/v uranyl acetate for 1 min. The stained exosome samples were examined using H‐7600 TEM (Hitachi, Japan) at an acceleration voltage of 80 kV. Cell samples were pelleted and fixed for 1 h in 2.5% glutaraldehyde in 0.1 m phosphate buffer at room temperature. The fixed samples were embedded in resin, thin sectioned, and stained with uranyl acetate.

*Coimmunoprecipitation*: Cells were lysed with lysis buffer containing 2.0 × 10^−2^ m Tris‐HCl, 0.15 m NaCl, 0.5% Nonidet P‐40, 2.0 × 10^−3^ m ethylenediaminetetraacetic acid (EDTA), 5.0 × 10^−2^ m dithiothreitol (DTT), 1.0 × 10^−3^ m NaF, 1.0 × 10^−3^ m phenylmethanesulfonyl fluoride (PMSF), and 1x protease inhibitor cocktail. Lysates were clarified by centrifugation at 10 000 g for 10 min at 4 °C. The supernatants were then incubated with anti‐mTOR antibody or control IgG followed by precipitation with protein G conjugated agarose beads. Beads were washed four times with lysis buffer and boiled with 1x SDS sample for 5 min. The precipitated proteins were analyzed by western blotting.

*Statistical Analysis*: Statistical analyses were performed using the GraphPad Prism 6.0 software. *P* value was determined by the Student's *t*‐test for two‐group, or one‐way ANOVA test for multiple group comparison. *P* < 0.05 is considered significant. All experiments were repeated at least three times. Quantitative data were expressed as mean ± standard deviation (S.D.)

## Conflict of Interest

The authors declare no conflict of interest.

## Supporting information

Supplementary

Supplementary

Supplementary

Supplementary
