# Supplementary material for: Exosome Release Is Regulated by mTORC1
Source: Adv Sci (Weinh). 2018 Dec 11;6(3):1801313. doi: 10.1002/advs.201801313 (PMC6364500; doi:10.1002/advs.201801313)
Supplement: Supplementary file 1 — Supplementary [file ADVS-6-1801313-s002.pdf]

Copyright WILEY-VCH Verlag GmbH & Co. KGaA, 69469 Weinheim, Germany, 2018.

ADVANCED SCIENCE
Open Access

Supporting Information

for *Adv. Sci.,* DOI: 10.1002/advs.201801313

Exosome Release Is Regulated by mTORC1

Wenchong Zou, Mingqiang Lai, Yue Zhang, Lei Zheng, Zhe Xing, Ting Li, Zhipeng Zou, Qiancheng Song, Xiaoyang Zhao, Laixin Xia, Jian Yang, Anling Liu, Han Zhang, Zhong-Kai Cui, Yu Jiang,* and Xiaochun Bai**

Supporting Information

Exosome Release is Regulated by mTORC1

Wenchong Zou, Mingqiang Lai, Yue Zhang, Lei Zheng, Zhe Xing, Ting Li, Zhipeng Zou, Qiancheng Song, Xiaoyang Zhao, Laixin Xia, Jian Yang, Anling Liu, Han Zhang, Zhong-Kai Cui*, Yu Jiang* and Xiaochun Bai*

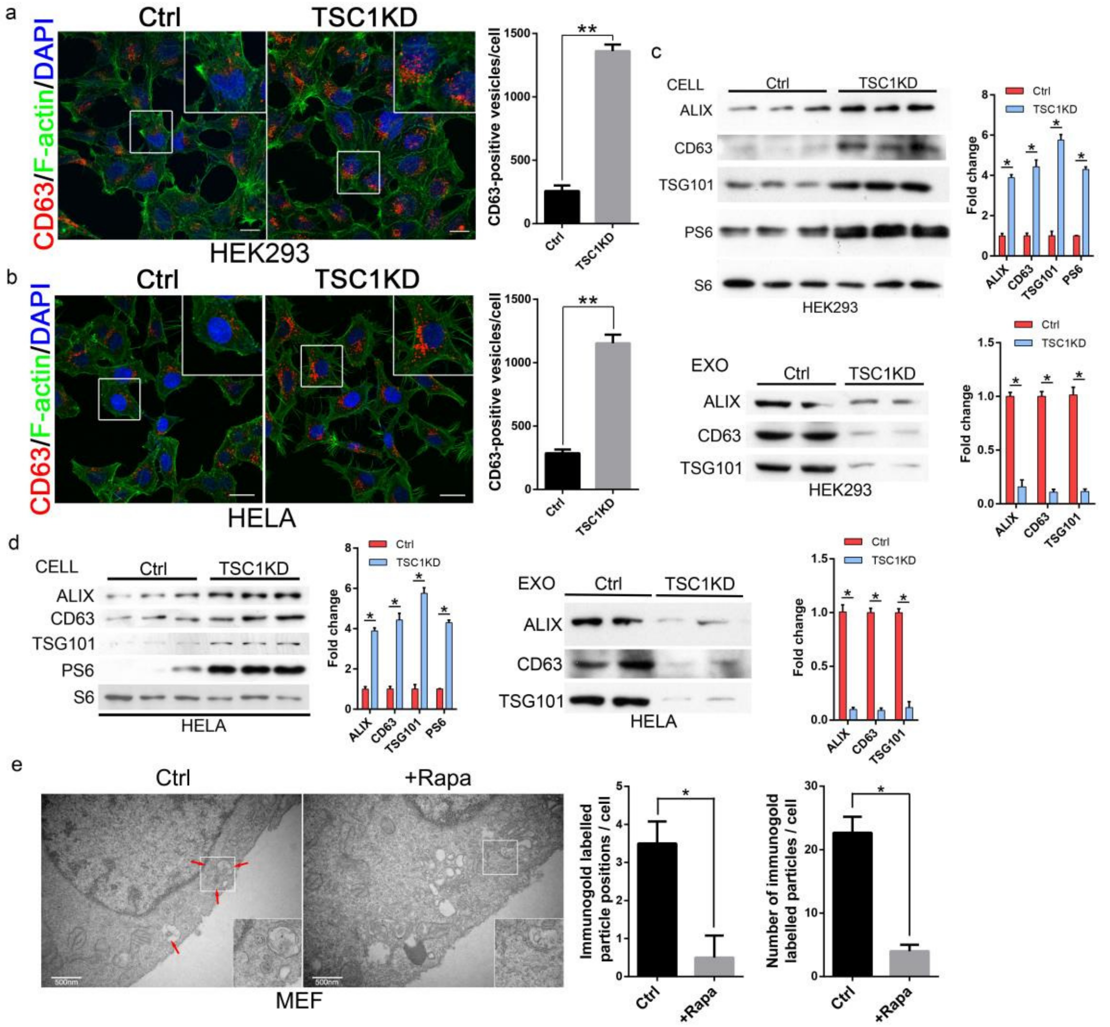

Figure S1. Activation of mTORC1 prevents exosome release in HEK293 and Hela cells. (a,b) HEK293 (a) and Hela (b) cells expressing TSC1 specific siRNA or scrambled siRNA were fixed and stained with anti- CD63 (red), phalloidin (green) and DAPI (blue), and imaged by confocal microscopy. Scale bars, 20 μm. The numbers of CD63-positive vesicles in TSC1

knockdown (KD) and control cells were determined and expressed as mean ± s.d. (right panels). A total of 60 randomly selected cells from three independent experiments was analyzed. **P<0.01. (c,d) Cell lysates and exosomes isolated from culture media of TSC1 KD HEK293 (c), Hela (d) and their respective control cells were analyzed by western blot for the levels of exosome marker proteins. Western blots were quantified. (e) Transmission electron microscopy analysis of MEF cells treated with rapamycin (100 nM) or vehicle control for 24 h. The immunogold labelled particles in the cell were marked by red arrows. Scale bars, 500 nm.

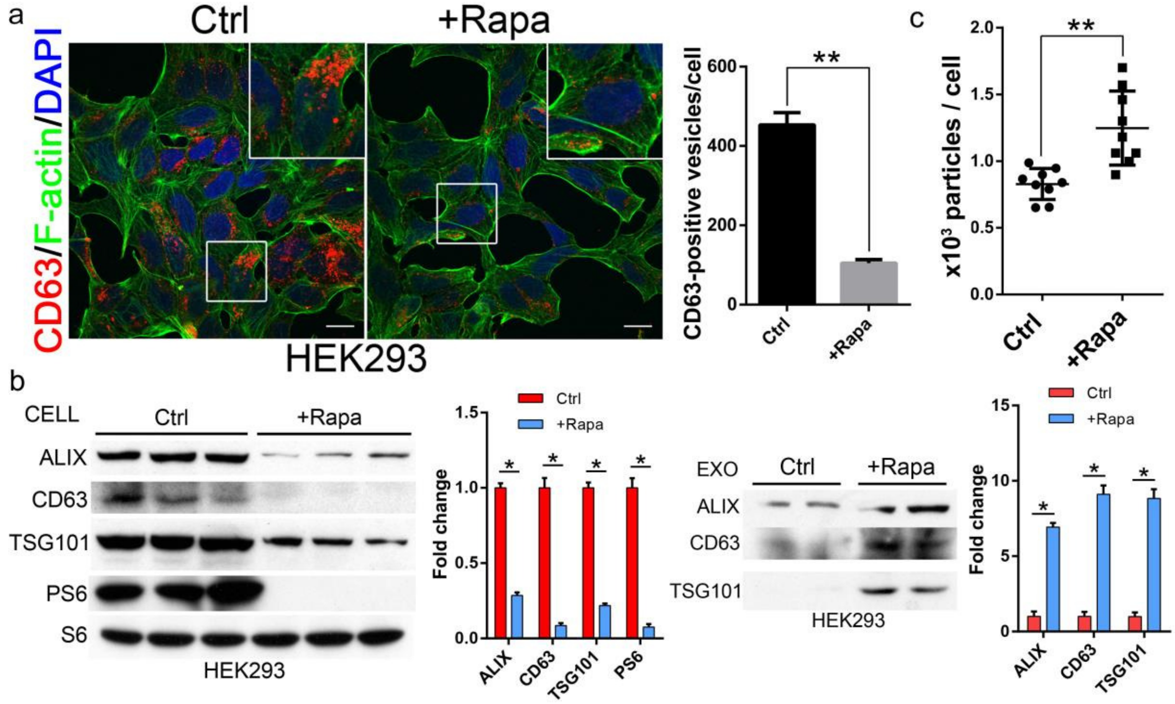

Figure S2. Rapamycin stimulates exosome release in HEK293 cells. HEK293 cells were treated with rapamycin or vehicle control for 24 hr. Cells were fixed and stained with anti‐ CD63 (red), phalloidin (green) and DAPI (blue), and imaged by confocal microscopy. Scale bars, 20 µm. (a) The amounts of CD63-positive vesicles in the cells were quantified and shown as mean ± s.d. (right panel). A total of 60 randomly selected cells from three independent experiments was analyzed. (b) The levels of exosome marker proteins in cell

lysates and exosomes isolated from culture media were assessed by western blot. (c) Western blots were quantified. The amounts of exosomes isolated from culture media were determined by NTA and presented as the mean ± s.d. Data are from three independent experiments. **P < 0.01.

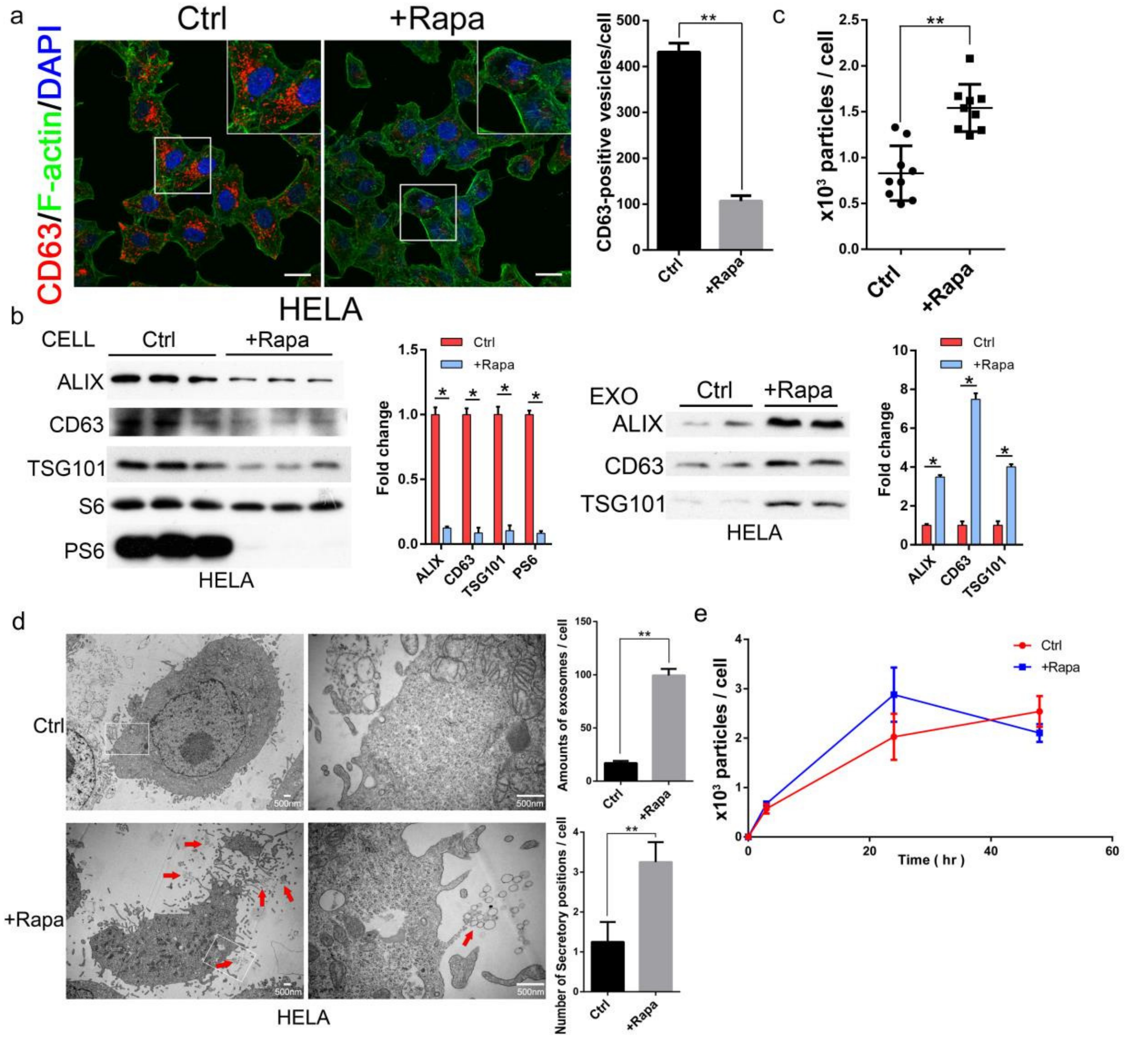

Figure S3. Rapamycin stimulates exosome releasein Hela cells. Hela cells were treated with rapamycin or vehicle control for 24 hr. Cells were fixed and stained with anti- CD63 (red), phalloidin (green) and DAPI (blue), and imaged by confocal microscopy. Scale bars, 20 µm. (a) The amounts of CD63-positive vesicles in the cells were quantified and shown as mean ±

s.d. (right panel). A total of 60 randomly selected cells from three independent experiments was analyzed. (b) The levels of exosome marker proteins in cell lysates and exosomes isolated from culture media were assessed by western blot. Western blots were quantified. (c) The amounts of exosomes isolated from culture media were determined by NTA and presented as the mean ± s.d. Data are from three independent experiments. **P < 0.01. (d) Transmission electron microscopy analysis of Hela cells treated with rapamycin (100 nM) or vehicle control for 24 h. The released exosomes in the extracellular space were marked by red arrows. Right panels are quantitative presentations of the numbers of exosomes in the extracellular space. Scale bars, 500 nm (e) The amounts of exosomes isolated from culture media of Hela cells treated with rapamycin (100 nM) or vehicle control for indicated times were assessed by NTA. ** P < 0.01.

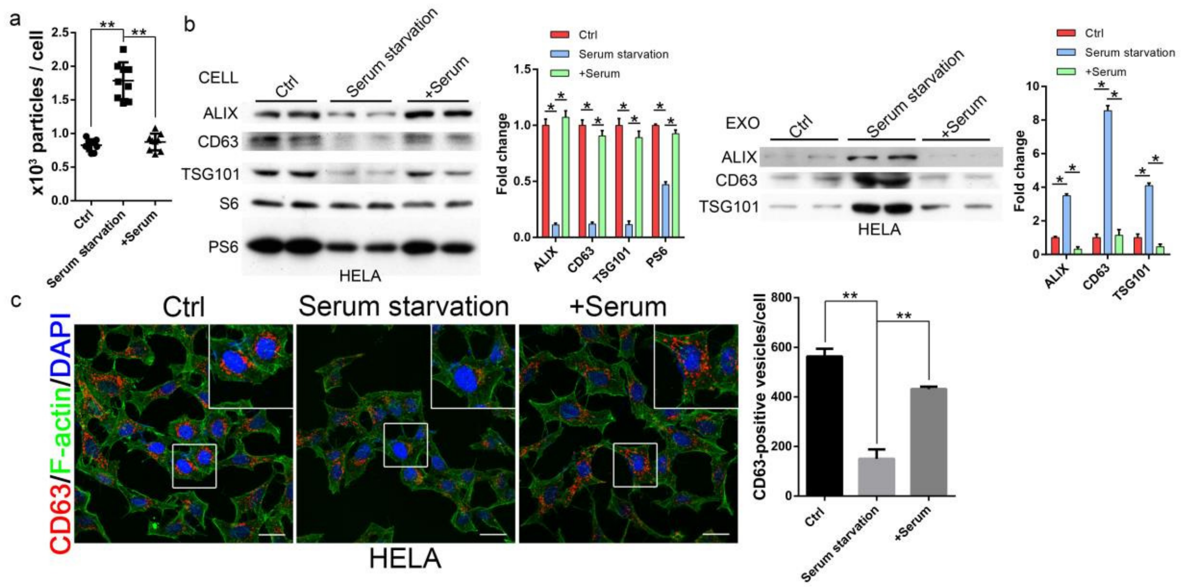

Figure S4. Amino acid or serum deprivation stimulates exosome release in Hela cells. Hela cells were grown in serum-free medium for 16 hr before being shifted to serum-containing medium for additional 16 hr. Cells and media were collected before and after the shift. (a) The numbers of exosomes isolated from culture media were assessed by NTA. (b) The levels of exosome marker proteins in cell lysates were determined by western blot. Western blots were

quantified. (c) Cells were stained with anti‑ CD63 (red), phalloidin (green) and DAPI (blue), and imaged by confocal microscopy. Scale bars, 20 μm. The numbers of CD63-positive vesicles in the cells were determined and expressed mean ± s.d. A total of 60 randomly selected cells from three independent experiments was analyzed. **P < 0.01.

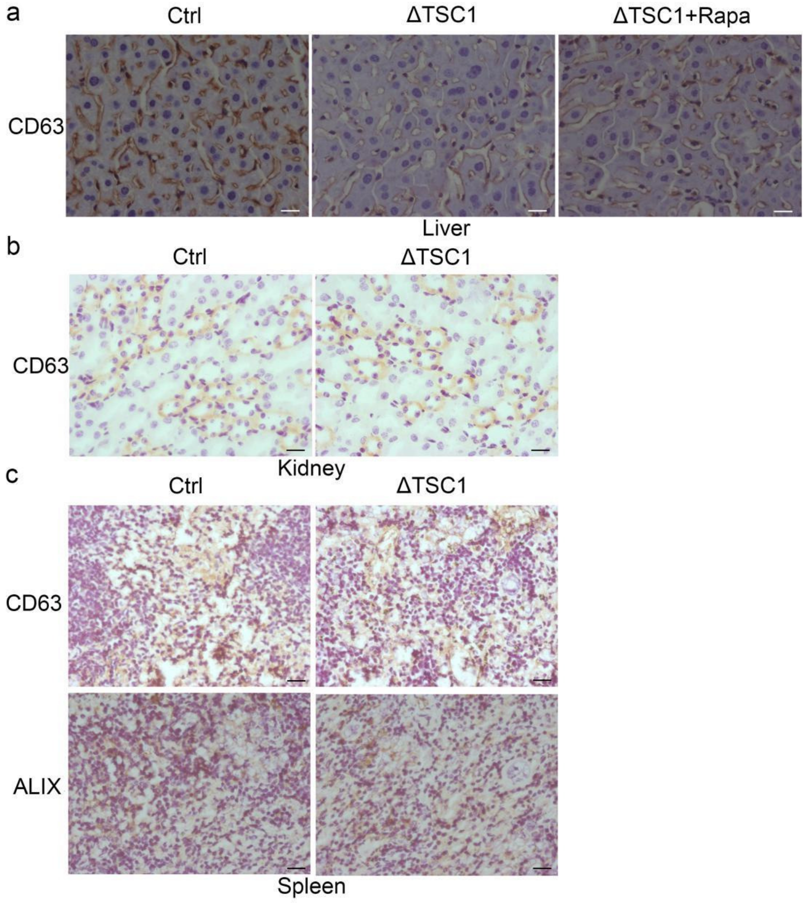

Figure S5. mTORC1 inhibits exosome release in mouse liver. Three-month-old mice (n = 5) with liver cell-specific TSC1 deletion (TSC1KO) were administrated with rapamycin (2.5 mg/kg/d) or drug vehicle control for 2 months. The animals were sacrificed and tissues collected, fixed and stained with anti-CD63 or Alix antibody for immunohistochemical analysis. (a) liver, (b) kidney, (c) spleen. Scale bars, 20 μm.

Movie S1. Rapamycin stimulates exosome release. Hela cells expressing GFP-CD63 were treated with rapamycin (100 nM). The secretion of GFP-CD63-positive exosomes from the cells during the first 2.5 h following addition of the drug was recorded by fluorescent microscopy. Scale bars, 20 µm.

Movie S2. Rapamycin stimulates exosome release. Hela cells expressing GFP-CD63 were treated with vehicle control (PBS). The secretion of GFP-CD63-positive exosomes from the cells during the first 2.5 h following addition of vehicle control was recorded by fluorescent microscopy. Scale bars, 20 µm.

Table S1. Up- or down-regulated proteins in exosomes from Hela cells treated with rapamycin (100 nM) for 24 h.
